# Uptake of Chlorin e_6_ Photosensitizer by Polystyrene-Diphenyloxazole-Poly(N-Isopropylacrylamide) Hybrid Nanosystem Studied by Electronic Excitation Energy Transfer

**DOI:** 10.1186/s11671-018-2584-4

**Published:** 2018-05-31

**Authors:** M. Yu. Losytskyy, L. O. Vretik, N. V. Kutsevol, O. A. Nikolaeva, V. M. Yashchuk

**Affiliations:** 1 Faculty of Physics, Taras Shevchenko National University of Kyiv, Volodymyrs’ka Str., 64/13, Kyiv, 01601 Ukraine; 2 Faculty of Chemistry, Taras Shevchenko National University of Kyiv, Volodymyrs’ka Str., 64/13, Kyiv, 01601 Ukraine

**Keywords:** Styrene nanoparticles, Stimuli-responsive materials, PNIPAM, Diphenyloxazole, Conformation transition, Chlorin e_6_, Electronic excitation energy transfer, Radiodynamic therapy

## Abstract

Polystyrene (PS)-diphenyloxazole (PPO) nanoparticles with attached cross-linked poly-N-isopropylacrylamide (PNIPAM) chains were obtained resulting in PS-PPO-PNIPAM hybrid nanosystems (NS). Fluorescence spectra of chlorin e_6_ added to PS-PPO-PNIPAM hybrid NS revealed electronic excitation energy transfer (EEET) from PS matrix and encapsulated PPO to chlorin e_6_. EEET efficiency increased strongly during 1 h after chlorin e_6_ addition, indicating that uptake of chlorin e_6_ by PNIPAM part of hybrid NS still proceeds during this time. Heating of PS-PPO-PNIPAM-chlorin e_6_ NS from 21 to 39 °C results in an enhancement of EEET efficiency; this is consistent with PNIPAM conformation transition that reduces the distance between PS-PPO donors and chlorin e_6_ acceptors. Meanwhile, a relatively small part of chlorin e_6_ present in the solution is bound by PNIPAM; thus, further studies in this direction are necessary.

## Background

The main disadvantage of the photodynamic therapy of cancer is the low depth of the excitation beam penetration into the tissue [1]. Thus, radiodynamic therapy approach to cancer treatment (where sensitizer could be efficiently excited with the X-rays able to penetrate deep into the body) was proposed [2] and is intensively studied in the last years [3–5]; one of the research scopes is the development of sensitizers that generate singlet oxygen upon X-ray excitation [5–8]. The key process in such X-ray sensitizer is the electronic excitation energy transfer (EEET) between its scintillating and sensitizing components [2, 6, 9–11]. Another important component of the mentioned X-ray sensitizer is the way of keeping scintillating and sensitizing parts together at the distance optimal for EEET; chemical conjugation [2, 3], electrostatic attraction [8, 12], surfactant [11], or polymer shell [4] could be mentioned. Earlier, in the frames of designing nanosystems (NS) for X-ray excited sensitizing of singlet oxygen, we studied EEET in polystyrene (PS)-diphenyloxazole (PPO)-chlorin e_6_ NS, where photosensitizer chlorin e_6_ was bound to PS-PPO nanoparticle (which can be used as scintillator [13, 14]) via surfactant (sodium dodecylsulphate) shell [15].

Poly(N-isopropylacrylamide) (PNIPAM) belongs to stimuli-responsive materials that change their properties in response to internal or external stimulus [16]. Linear PNIPAM is known to undergo conformational transition upon heating, i.e., the polymer shrinks (due to becoming hydrophobic and thus expelling water molecules) at the temperatures over the lower critical solution temperature (LCST) that equals 32 °C for linear PNIPAM [17]. Considerable decrease in the PNIPAM shell width upon temperature transition over LCST was shown for PS-PNIPAM nanoparticles in [17]. At the same time, for the PNIPAM chains conjugated to dextran, the temperature of conformation transition was shown to be 2–4 °C higher as compared to linear PNIPAM of similar molecular weight and polydispersity due to the steric interaction between PNIPAM chains hindering the conformation transition [18].

**Fig. 1:**
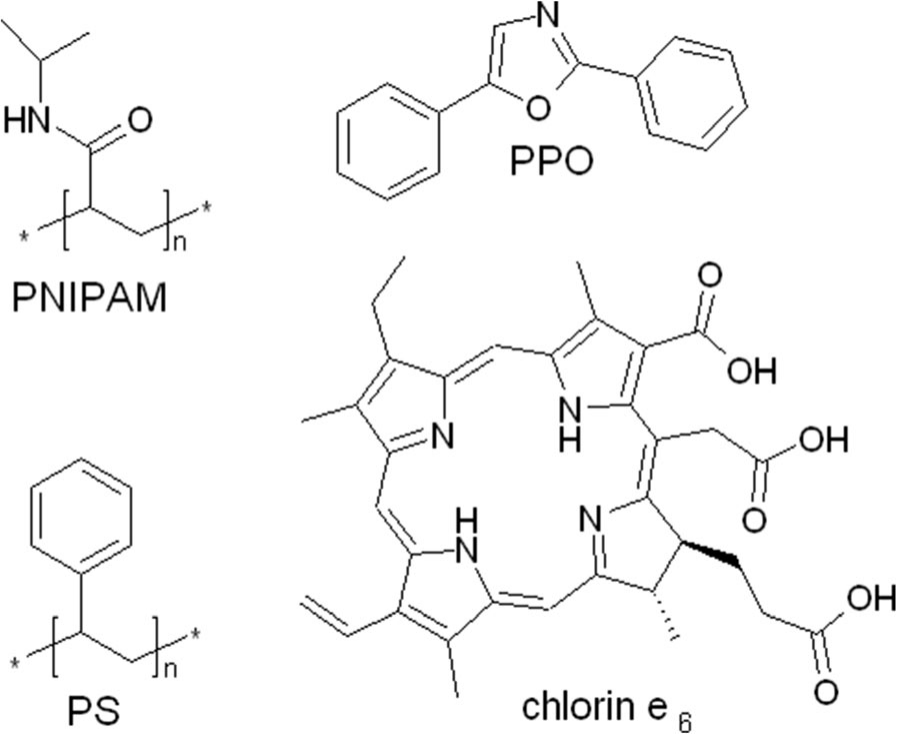
Structures of the components of the PS-PPO-PNIPAM hybrid nanosystem and chlorin e_6_

**Fig. 2:**
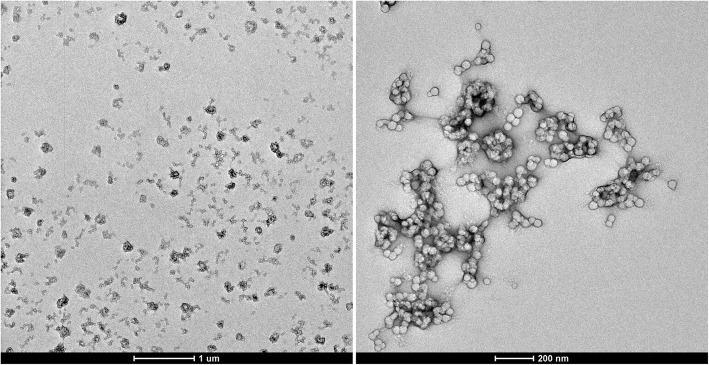
TEM images of the obtained PS-PPO-PNIPAM hybrid nanosystems with lower (left) and higher (right) magnification

In this work, PS-PPO nanoparticles covered by cross-linked PNIPAM shell (Figs. 1 and 2) were obtained resulting in PS-PPO-PNIPAM hybrid NS. The possibility of using cross-linked PNIPAM for attaching chlorin e_6_ sensitizer to PS-PPO NP scintillator was studied. This polymer has the conformational transition at physiological temperatures (for cross-linked PNIPAM, LCST should be higher than 32 °C). Thus, shrinking of cross-linked PNIPAM network following heating under excitation could result in decrease of the distance between PS-PPO donor and chlorin e_6_ acceptor that would increase the efficiency of EEET and thus the efficiency of tumor destruction.

## Experimental

### Materials

Styrene (ST, Ukraine) of p.a. quality was purified via standard method directly before polymerization. N-Isopropylacrylamide (NIPAM, Sigma-Aldrich Inc.), N,N′-methylenebisacrylamide (BIS, Sigma-Aldrich Inc.), potassium persulfate K_2_S_2_O_8_ (KPS, Ukraine), sodium phosphate monobasic dehydrate NaH_2_PO_4_ × 2H_2_O (Ukraine), and anionic surfactant sodium dodecyl sulfate (SDS, Sigma-Aldrich Inc.) were of reagent grade and used without further purification. Chlorin e_6_ (Frontier Scientific Inc.) was kindly provided by T.Y. Ohulchanskyy (Institute for Lasers, Photonics and Biophotonics at the State University of New York at Buffalo). Fifty millimolars of TRIS-HCl buffer (pH 7.2) was used as solvent.

### Synthesis and Characterization of Nanosystems

Polystyrene-poly(N-isopropylacrylamide) hybrid nanosystems, doped with PPO (PS-PPO-PNIPAM hybrid NS), were synthesized as follows. First, PS-co-PNIPAM core nanoparticles doped with PPO were prepared by microemulsion polymerization [13, 14, 19, 20]. Briefly, 0.2 g of NIPAM, 0.2 g of sodium dodecylsulphate, and 0.01 g of NaH_2_PO_4_ × H_2_O were dissolved in 90 g of H_2_O. Then, 0.09 g of PPO was dissolved in 1.8 g of styrene and the obtained mixture was added dropwise during 30 min, too. The mixture was stirred at 700 rpm, and Ar was bubbled into the mixture for 30 min. After the temperature increased to 70 °C, 0.01 g of K_2_S_2_O_8_ dissolved in 1 ml of H_2_O was injected to initiate the polymerization. Secondly, PNIPAM shell layer was fabricated after 4 h heating at 70 °C. For this purpose, aqueous solution of monomer NIPAM (0.69 g) and cross-linker N,N′-methylenebisacrylamide (BIS) (0.06 g) were added into the reactor using a syringe. The reaction was allowed to continue for 3 h at 70 °C and additional 1 h at 90 °C. The mixture was cooled to room temperature and dialyzed during 48 h using cellulose membrane with MWCO 3500 Da.

Transition electron microscopy (TEM) images of the obtained nanosystems are presented in Fig. 2. For the sample preparation, 400 mesh Cu grids with plain carbon film were rendered hydrophilic by a glow discharge treatment (Elmo, Cordouan Technologies Bordeaux France). A 5-μl drop was deposited and let adsorbed for 1 min, then the excess of solution was removed with a piece of filter paper. The observations of the PS-PPO-PNIPAM nanosystems were carried on two TEMs, Tecnai G2 or CM12 (FEI, Eindhoven, Netherlands), and the images were acquired with a ssCCD Eagle camera on the Tecnai and a Megaview SIS Camera on the CM12. It is seen from Fig. 2 that the obtained hybrid nanosystems consist of several bound spherical PS-PPO NP; we believe that they are bound by cross-linked PNIPAM polymer net. Thus, PS-PPO-PNIPAM hybrid nanosystems were obtained.

### Spectral Measurements and Sample Preparation

Absorption spectra were measured using a Specord M40 spectrophotometer (Carl Zeiss, Germany). Fluorescence excitation and emission spectra were registered with the help of a Cary Eclipse fluorescent spectrophotometer (Varian, Australia). Absorption and fluorescence measurements were performed in 1 × 1 cm quartz cell at room temperature. Fifty millimolars of TRIS-HCl buffer, pH 7.2, was used as a solvent. For spectral measurements, the obtained solution of PS-PPO-PNIPAM hybrid NS was dissolved 100 times in buffer. Stock solution of chlorin e_6_ at the concentration 10 mM was prepared in DMF and further diluted in buffer to 1 mM concentration. Small aliquot of this 1 mM solution of chlorin e_6_ was then added to 100 times dissolved buffer solution of NS; the final concentration of chlorin e_6_ was 2 μM, and DMF admixture was thus 0.02%. Fluorescence excitation and emission spectra of PS-PPO-PNIPAM hybrid NS solution with chlorin e_6_ was measured in 0, 5, 10, 20, 40, 60, 80, and 100 min after the addition of chlorin e_6_ to the solution of PS-PPO-PNIPAM hybrid NS. In nearly 80 min, the saturation was reached.

For temperature-dependent measurements, solution of PS-PPO-PNIPAM hybrid NS in the presence of chlorin e_6_ was placed into the thermostatted cell holder (*T* = 23 °C). In 88 min after the preparation of the sample, after the uptake of chlorin e_6_ by PS-PPO-PNIPAM hybrid NS reached saturation, water flow from the water bath was switched on heating the sample to 39 °C (this temperature should exceed LCST for the cross-linked PNIPAM). Fluorescence excitation spectra of chlorin e_6_ (emission at 680 nm) were then measured in different time intervals after the heating was started. Experimental results state that the conformation transition starts in about 3 min after the heating was started. It should be mentioned that in about 18 min after the heating was started, coagulation of the NS occurred giving macroscopic clots (which however disappear after the solution was cooled down, thus coagulation is reversible). Temperature experiment was performed three times, and similar tendencies were obtained.

## Results and Discussion

**Fig. 3:**
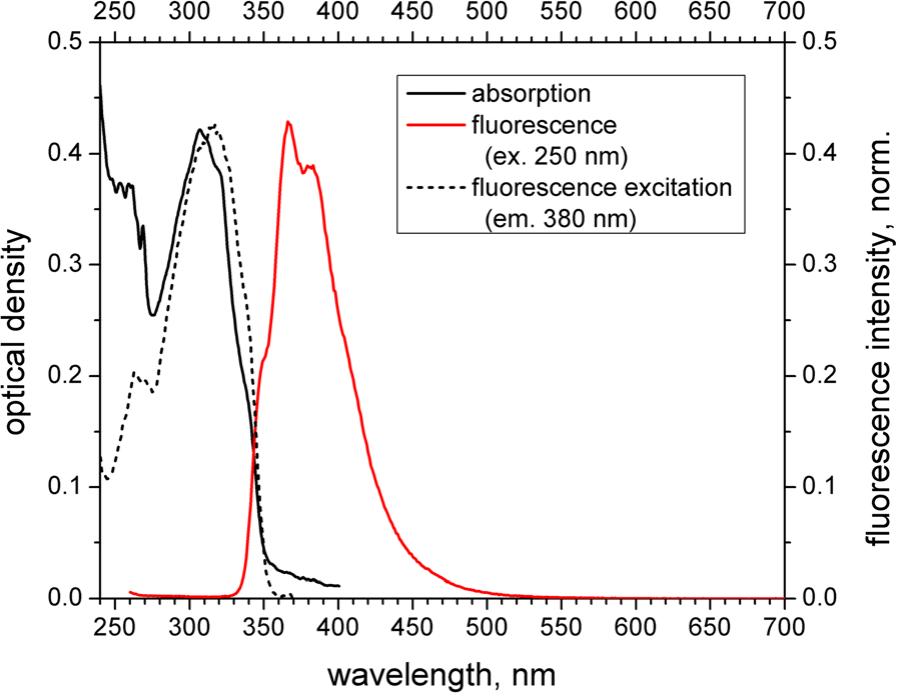
Absorption (black solid line), fluorescence excitation (emission at 380 nm, normalized; black short-dashed line), and emission (excitation at 250 nm, normalized; red solid line) spectra of the obtained PS-PPO-PNIPAM hybrid nanosystems in 50 mM Tris-HCl buffer (pH 7.2)

Absorption, fluorescence excitation, and fluorescence emission spectra of the obtained PS-PPO-PNIPAM hybrid nanosystems in 50 mM Tris-HCl buffer (pH 7.2) are presented in Fig. 3. Absorption spectrum contains the bands corresponding to styrene (maximum near 260 nm) and PPO (maximum near 307 nm). At the same time, fluorescence emission spectrum upon excitation at 250 nm (the range of styrene absorption) resulted in the spectrum that belongs exclusively to PPO (with maximum at 367 nm), while the emission of styrene (due at 307 nm [15]) was not observed. Thus, EEET from styrene to incorporated PPO is near to complete. EEET is also supported by the excitation spectrum of PPO emission (380 nm) where styrene band near 260 nm is clearly observed (Fig. 3). It should be added that clear vibronic structure could be observed in PPO emission spectrum (which is not observed for the PPO water solution [14]) that additionally points to the PPO incorporation into the PS matrix.

**Fig. 4:**
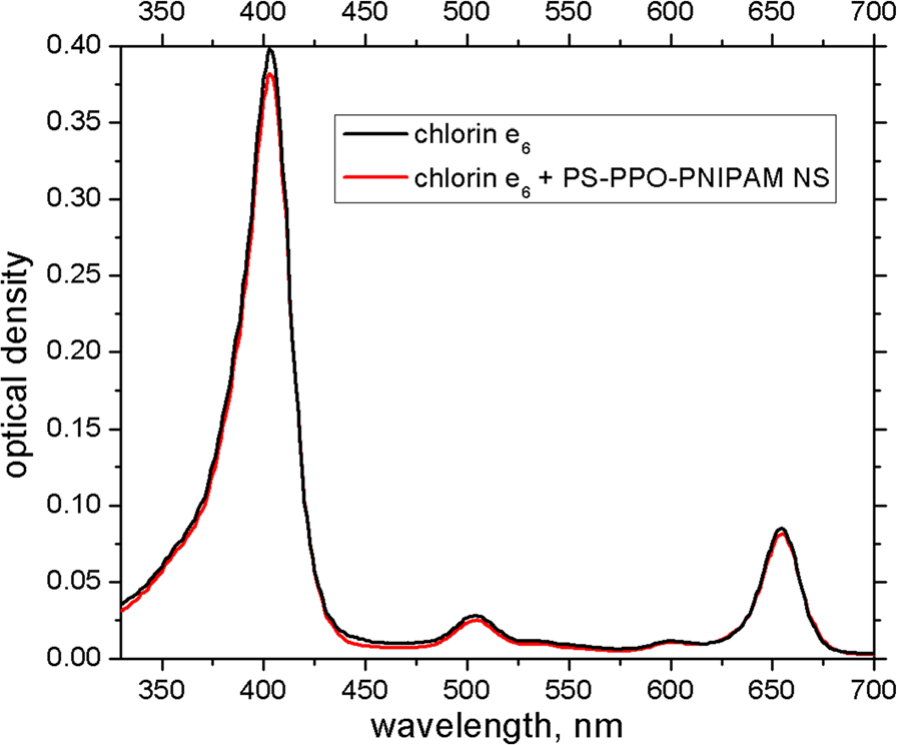
Absorption spectra of chlorin e_6_ (2 μM) free (black line) and in the presence of PS-PPO-PNIPAM hybrid nanosystems (red line) in 50 mM Tris-HCl buffer, pH 7.2

Further, to the solution of the obtained PS-PPO-PNIPAM hybrid nanosystems in 50 mM Tris-HCl buffer (pH 7.2), the photosensitizer chlorin e_6_ was added. Absorption spectrum of chlorin e_6_ shows almost no change in the presence of hybrid nanosystems as compared to buffer solution, except small decrease of the optical density in the maxima (Fig. 4).

**Fig. 5:**
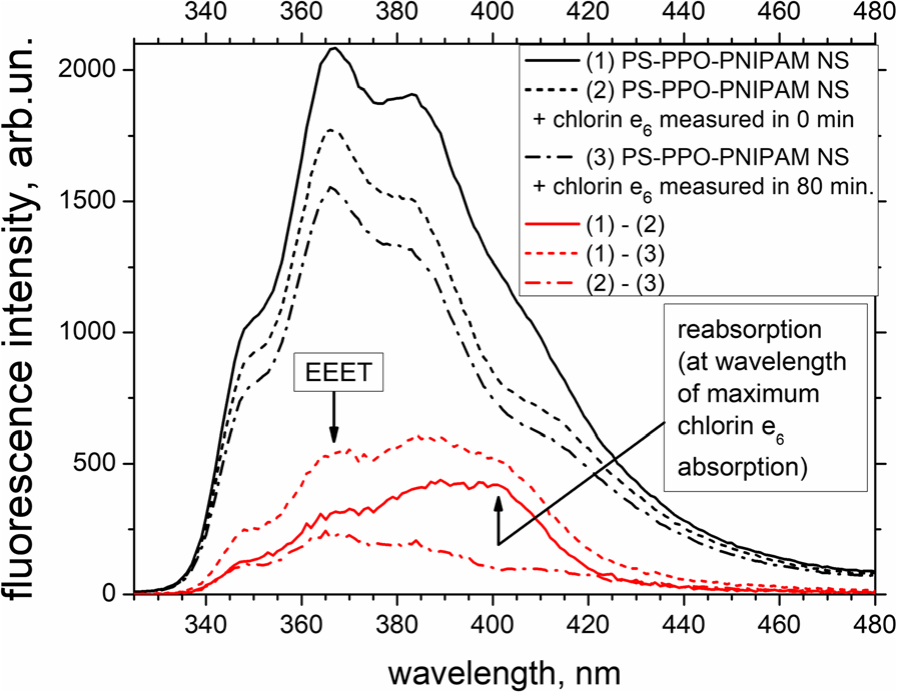
Fluorescence spectra of PS-PPO-PNIPAM hybrid NS free (black solid line) and upon addition of chlorin e_6_ measured in 0 min (black short-dashed line) and in 80 min (black dash-dotted line). The excitation wavelength is 250 nm. Fifty millimolars of Tris-HCl buffer, pH 7.2, is used as solvent. Differences of the spectra (solid, short-dashed and dash-dotted red lines) indicate the contribution of reabsorption and electronic excitation energy transfer (EEET) to emission quenching

At the same time, effect of the addition of chlorin e_6_ to PS-PPO-PNIPAM hybrid NS on fluorescence spectra is much more noticeable as compared to absorption ones. First of all, the presence of chlorin e_6_ leads to the quenching of PPO fluorescent emission of PS-PPO-PNIPAM hybrid NS, and this quenching does enhance during about an hour (Fig. 5). Generally, such quenching could be due to either EEET (that would lead to the quenching of donor emission at all emission wavelengths) or reabsorption (that would lead to the quenching of donor emission at the wavelengths of the acceptor absorption). Differences of PPO emission spectra (Fig. 5) show that immediately after chlorin e_6_ addition, the contribution of reabsorption to PPO emission quenching is significant (but EEET also takes place). At the same time, the contribution of reabsorption further decreases with time and this of EEET grows.

**Fig. 6:**
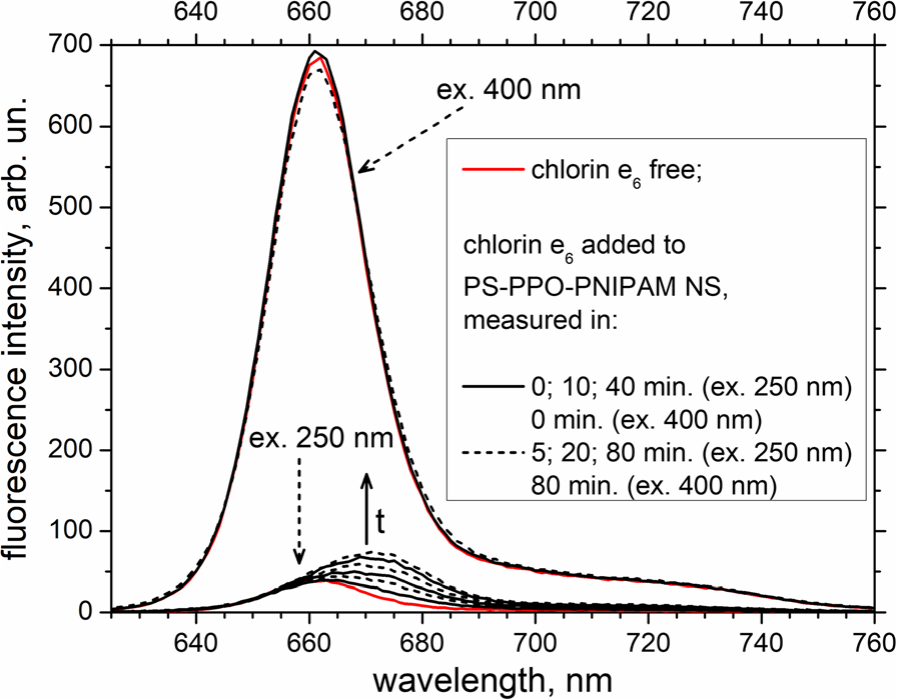
Fluorescence spectra of chlorin e_6_ free and added to PS-PPO-PNIPAM hybrid NS (measured in 0, 5, 10, 20, 40, and 80 min after addition). Excitation wavelengths are 250 and 400 nm; 50 mM Tris-HCl buffer, pH 7.2, was used as solvent. Short-dashed arrows indicate spectra excited at 250 and 400 nm. Solid arrow indicates the increasing time (t) after the addition of chlorin e_6_ for the spectra excited at 250 nm

Another effect of addition of chlorin e_6_ to PS-PPO-PNIPAM hybrid NS is the appearing of chlorin e_6_ emission (upon excitation of PS at 250 nm) that is shifted to the long-wavelength region as compared to the one of free chlorin e_6_; both intensity and shift do increase with time and reach saturation in about an hour (Fig. 6). This shift points to the influence of the PNIPAM surrounding on the bound molecules of chlorin e_6_. At the same time, emission of chlorin e_6_ upon excitation to its own absorption (at 400 nm) is only slightly changed in the presence of PS-PPO-PNIPAM NS. Together with the small change in chlorin e_6_ absorption (Fig. 4), this means that only small part of chlorin e_6_ molecules is bound to PS-PPO-PNIPAM NS.

**Fig. 7:**
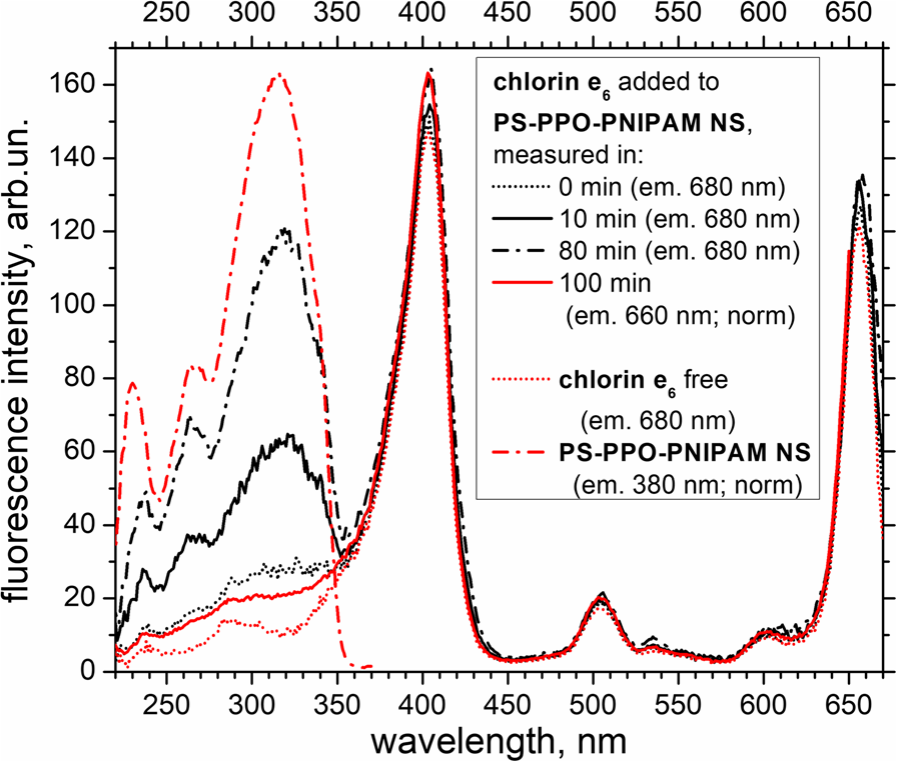
Fluorescence excitation spectra of chlorin e_6_ free and added to PS-PPO-PNIPAM hybrid NS (measured in 0, 10, 80, and 100 min after addition), and PS-PPO-PNIPAM hybrid NS itself. Emission wavelengths for chlorin e_6_ are 660 nm (normalized intensity) and 680 nm. Emission wavelength for PS-PPO-PNIPAM NS is 380 nm (normalized intensity). Fifty millimolars of Tris-HCl buffer, pH 7.2, is used as solvent

Finally, the PS-PPO band appears in excitation spectrum of chlorin e_6_ added to PS-PPO-PNIPAM hybrid NS (emission at 680 nm, where the contribution of the PNIPAM-bound chlorin e_6_ to the total emission of chlorin e_6_ is close to maximum). This band is weak at first, but further, its intensity strongly increases with time (Fig. 7). At the same time, this PS-PPO band is very weak in the excitation spectrum of chlorin e_6_ upon emission at 660 nm (i.e., at maximum of the free chlorin e_6_ spectrum) even after 100 min after the addition of chlorin e_6_.

Thus, chlorin e_6_ binds to PS-PPO-PNIPAM hybrid NS that causes EEET from PS matrix and encapsulated PPO to chlorin e_6_. EEET efficiency enhances with time (during about an hour after chlorin e_6_ addition), indicating that uptake of chlorin e_6_ by PNIPAM network of PS-PPO-PNIPAM hybrid NS still proceeds during this time. At the same time, a relatively small part of chlorin e_6_ present in the solution is bound by PNIPAM.

It should be also mentioned that PS-PPO NP were shown to emit the fluorescence of PPO [14] or attached porphyrin [13] when excited with X-rays. Thus, EEET from PS to chlorin e_6_ observed in PS-PPO-PNIPAM hybrid NS under the UV excitation of PS means that the energy of X-rays could be also transferred to chlorin e_6_ in the studied NS. At the same time, rather high concentration of PS-PPO NP is required for the direct observation of X-ray stimulated emission of such NP [13, 14]. One of the ways to increase the sensitivity of such NS to X-rays could be the addition of components containing heavy atoms.

PNIPAM is known to experience the conformation transition at LCST equal to 32 °C. For the cross-linked polymer, the LCST value should be still higher. If our PS-PPO-PNIPAM hybrid NS consists of cross-linked PNIPAM network surrounding PS-PPO NPs, and chlorin e_6_ is bound to PNIPAM network, we could expect the decrease of PPO-chlorin e_6_ distance and thus the increase of EEET efficiency upon heating the whole hybrid NS.

**Fig. 8:**
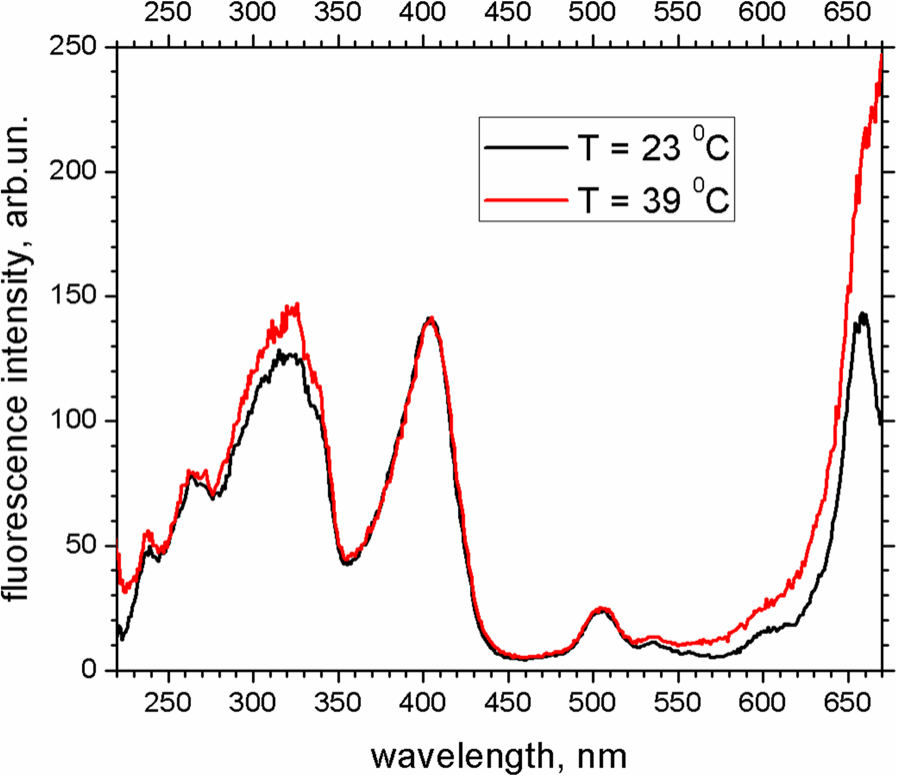
Fluorescence excitation spectra (normalized at 404 nm) of chlorin e_6_ added to PS-PPO-PNIPAM hybrid NS at 23 °C (black line) and at 39 °C (in 14 min after heating was switched on; red line). Emission wavelength 680 nm; 50 mM Tris-HCl buffer, pH 7.2, was used as solvent

**Fig. 9:**
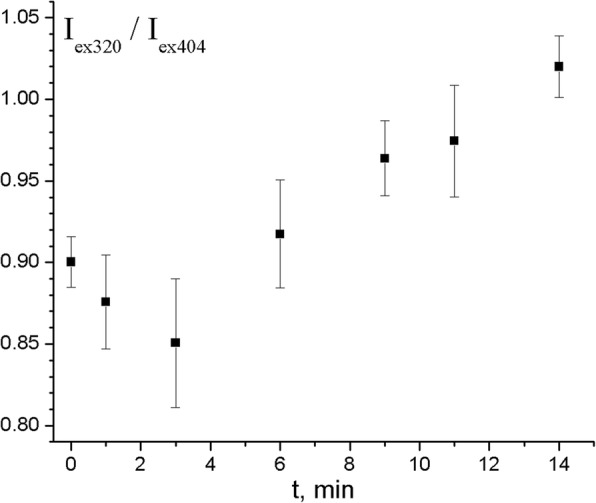
The dependence on the time after the heating to 39 °C was switched on of the ratio of the emission intensity of chlorin e_6_ added to PS-PPO-PNIPAM hybrid NS at 680 nm upon excitation at 320 nm (absorption by PPO with further EEET to chlorin e_6_) to that upon excitation at 404 nm (direct excitation of chlorin e_6_). Error bars account for the noise of the registered excitation spectra

To verify this idea, the effect of heating of PS-PPO-PNIPAM hybrid NS in the presence of chlorin e_6_ on fluorescence excitation spectra of chlorin e_6_ was studied. Heating experiment was performed three times; similar tendencies were demonstrated. Results of one of these experiments are presented in Figs. 8 and 9. Thus, the solution of PS-PPO-PNIPAM hybrid NS in the presence of chlorin e_6_ was heated to the temperature of 39 °C (this temperature should exceed LCST for the cross-linked PNIPAM); the heating started after the uptake of chlorin e_6_ by PS-PPO-PNIPAM hybrid NS reached saturation. During the heating, the ratio of chlorin e_6_ fluorescence intensities (emission at 680 nm) upon excitation at 320 nm (*I*_ex320_; that is chlorin e_6_ emission due to PPO-to-chlorin e_6_ EEET) and 404 nm (*I*_ex404_; that is chlorin e_6_ emission upon excitation to its own Soret band) was studied (Figs. 8 and 9); we believe this ratio to reflect the efficiency of PPO-to-chlorin e_6_ EEET. Thus, the first 3 min after heating started, *I*_ex320_/*I*_ex404_ decreased from 0.9 to 0.85, perhaps due to the decrease of the chlorin e_6_-to-PNIPAM binding affinity upon temperature increase. Further, the value of *I*_ex320_/*I*_ex404_ ratio increased up to 1.02, which was accompanied by the increased light scattering (Fig. 8; scattering is manifested as the intensity increase near 660 nm). This could be explained by conformation transition in PNIPAM that leads to reducing of cross-linked PNIPAM network volume. This causes the decrease of the distance between PS-PPO donor and chlorin e_6_ acceptor molecules bound to PNIPAM network and thus to increasing of EEET efficiency.

**Fig. 10:**
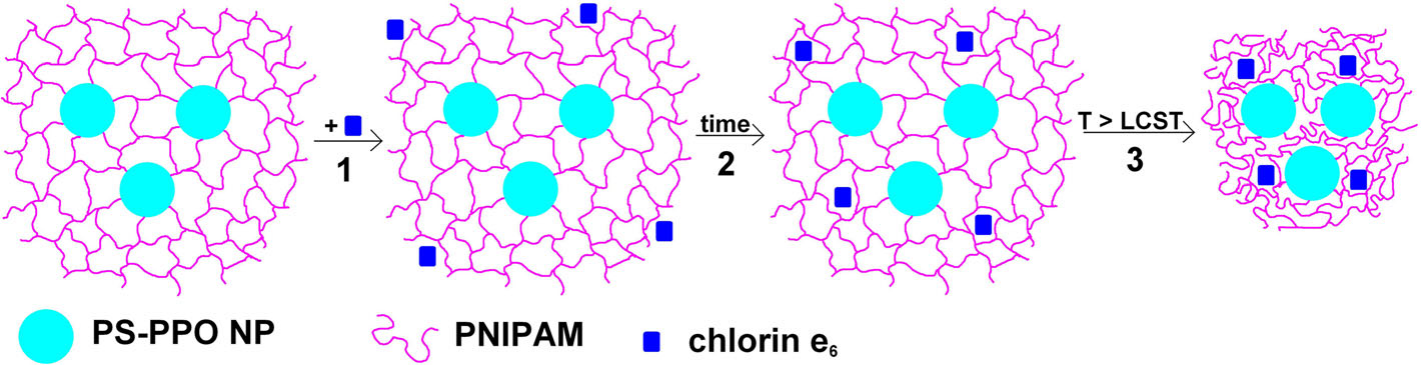
Proposed scheme of the processes in the solution of PS-PPO-PNIPAM hybrid NS in the presence of chlorin e_6_ upon addition of chlorin e_6_ (process 1), passing of about 1 h (process 2) and further heating from 23 to 39 °C (process 3)

In summary, processes in the solution of PS-PPO-PNIPAM hybrid NS in the presence of chlorin e_6_ addition could be described as follows. First, addition of chlorin e_6_ to the solution of PS-PPO-PNIPAM hybrid NS in buffer (Fig. 10, process 1) leads to the penetration of the small part of chlorin e_6_ into the PNIPAM network of the hybrid NS; this resulted in EEET from PS and PPO to chlorin e_6_. Such penetration proceeds during about an hour accompanied with the increase in EEET efficiency (Fig. 10, process 2). Further heating of the sample to the temperature exceeding LCST results in the conformation transition of the PNIPAM network that leads to the decrease of the distance between PS-PPO and chlorin e_6_ molecules, and thus to still higher increase of PS-PPO-to-chlorin e_6_ EEET efficiency (Fig. 10, process 3).

## Conclusions

The fluorescent study revealed the uptake of chlorin e_6_ by PS-PPO-PNIPAM hybrid NS as well as electronic excitation energy transfer from the PS matrix via encapsulated PPO to chlorin e_6_; uptake reached saturation in about an hour.

Heating of PS-PPO-PNIPAM-chlorin e_6_ NS from 21 to 39 °C results in enhancement of EEET efficiency; this is consistent with PNIPAM conformation transition that reduces the distance between PS-PPO donors and chlorin e_6_ acceptors.

Meanwhile, a relatively small part of chlorin e_6_ present in the solution is bound to PNIPAM; thus, further studies in this direction are necessary.

## Abbreviations

EEET: Electronic excitation energy transfer
LCST: Lower critical solution temperature
NS: Nanosystem
PNIPAM: Poly-N-isopropylacrylamide
PPO: Diphenyloxazole
PS: Polystyrene
TEM: Transmission electron microscopy
